# Electrohydrodynamic-Jet-Printed SnO_2_-TiO_2_-Composite-Based Microelectromechanical Systems Sensor with Enhanced Ethanol Detection

**DOI:** 10.3390/s24154866

**Published:** 2024-07-26

**Authors:** Danyang Wang, Dongqi Yu, Menghan Xu, Xue Chen, Jilin Gu, Lei Huang

**Affiliations:** 1 School of Physics and Electronic Technology, Liaoning Normal University, Dalian 116029, China; w_danyang@126.com (D.W.); dqyu@lnnu.edu.cn (D.Y.); lnnuxumenghan@lnnu.edu.cn (M.X.); 1430155786@qq.com (X.C.); 2 Research Center of Nano Science and Technology, College of Sciences, Shanghai University, Shanghai 200444, China

**Keywords:** gas sensor, ethanol, SnO_2_, TiO_2_, micro-electromechanical system

## Abstract

Ethanol sensors have found extensive applications across various industries, including the chemical, environmental, transportation, and healthcare sectors. With increasing demands for enhanced performance and reduced energy consumption, there is a growing need for developing new ethanol sensors. Micro-electromechanical system (MEMS) devices offer promising prospects in gas sensor applications due to their compact size, low power requirements, and seamless integration capabilities. In this study, SnO_2_-TiO_2_ nanocomposites with varying molar ratios of SnO_2_ and TiO_2_ were synthesized via ball milling and then printed on MEMS chips for ethanol sensing using electrohydrodynamic (EHD) printing. The study indicates that the two metal oxides dispersed evenly, resulting in a well-formed gas-sensitive film. The SnO_2_-TiO_2_ composite exhibits the best performance at a molar ratio of 1:1, with a response value of 25.6 to 50 ppm ethanol at 288 °C. This value is 7.2 times and 1.8 times higher than that of single SnO_2_ and TiO_2_ gas sensors, respectively. The enhanced gas sensitivity can be attributed to the increased surface reactive oxygen species and optimized material resistance resulting from the chemical and electronic effects of the composite.

## 1. Introduction

As an important organic compound, ethanol not only plays a key role as a solvent and organic synthesis reactant in traditional industries, but also has a wide range of applications in antifreeze, fuel, medical disinfection and other fields [1,2,3]. However, ethanol is flammable and can easily cause fires when exposed to high temperatures or flames. In addition, ethanol is easy to volatilize to form vapor and mix with air to form an explosive mixture [4,5,6]. Therefore, effective detection of ethanol vapor is particularly necessary. The resistance-based gas sensor is one of the research hotspots for fast ethanol detection. Among different sensing materials, metal oxide semiconductors (MOSs) can change the resistance in different kinds and concentrations of gases and have many advantages, such as high sensitivity, fast response, low cost, etc. However, traditional MOS-based sensors using electric heating wire suffer from high energy consumption and low response and consistency, necessitating the development of new materials and devices [7,8,9,10,11].

Micro-electro-mechanical systems (MEMS) devices have been attracting increasing attention in MOS gas sensors due to their low energy consumption, device miniaturization, and reduced device variation [12,13,14,15]. Most micro hot plate chips used contain substrate, microheater, and sensing electrodes. The sensitive MOS materials are then deposited on the surface of the micro hot plate for gas sensing. To date, there are three commonly used deposition methods for MEMS gas sensor gas-sensitive materials: dispensing, printing, and sputtering [16,17]. Among them, dispensing is mainly operated by a single dispenser, and its advantage lies in its lower cost and more porous gas-sensitive coating, which facilitates gas absorption reactions. However, it is very difficult to prepare the films over small MEMS chips using the dispenser technique. The recently developed electrohydrodynamic (EHD) printing technology is suitable for smaller chips and can effectively control print volume to maintain consistency, but it has high requirements for the printing slurry [18,19,20,21,22].

Among various MOS sensing materials, SnO_2_ has good stability and strong low-temperature working ability, while TiO_2_ has excellent stability and selectivity, but its application is limited by high working temperature and high resistance [23,24]. By compounding SnO_2_ and TiO_2_, the advantages of the two materials can be combined to form a material with good stability, low resistance and high selectivity [25,26,27,28,29]. The ceramic-based gas sensor composed of SnO_2_ and TiO_2_ boasts significant advantages and has been reported to detect triethylamine (TEA) using TiO_2_@SnO_2_. In the case of 5 ppm TEA gas, the response value of the TiO_2_@SnO_2_ sample at a working temperature of 240 °C reaches up to 21.6, which is three times higher than that of the TiO_2_ sample. Compared to TiO_2_, the response speed of the TiO_2_@SnO_2_ sensor is improved by 28%. However, there are relatively few reports on MEMS-based SnO_2_-TiO_2_ composite material sensors. With technological advancements in the future, their application prospects are worth anticipating [30].

The present work is motivated to fabricate an efficient and energy-saving MOS based MEMS sensor. Here, SnO_2_-TiO_2_ composite material was used to achieve good sensing performance and suitable resistance. Mechanical ball milling technique was applied to obtain uniformly dispersed SnO_2_-TiO_2_ nanocomposites slurry for the EHD printing. The detailed characterization of the MEMS sensors has been discussed. The gas-sensitive performances at different operating temperatures, humidities, and ethanol concentrations were studied. The ratios of SnO_2_-TiO_2_ were optimized to achieve good sensing performance. Finally, the promotional effect of SnO_2_-TiO_2_ ratios was discussed. This work provides ideas for designing efficient composite gas-sensitive materials and the fabrication of corresponding MEMS sensors. 

## 2. Experimental

### 2.1. Preparation of SnO_2_-TiO_2_ Nano-Paste and MEMS Chip

SnO_2_ (50–70 nm, 99.99%) was purchased from Shanghai Macklin Biochemical Technology Co., Ltd. (Shanghai, China); TiO_2_ (rutile type, 25 nm, 99.8%) was purchased from Shanghai Aladdin Biochemical Technology Co., Ltd. (Shanghai, China); ethylene glycol (AR), anhydrous ethanol (AR), anhydrous methanol (AR), and formaldehyde (AR) were purchased from Sinopharm Chemical Reagent Co., Ltd. (Shanghai, China); CO, NH_3_, and H_2_ were purchased from Shanghai Weichuang Standard Gas Analysis Technology Co., Ltd. (Shanghai, China). All chemicals were analytical grade chemicals from Sinopharm Chemical Reagent Company and were used without further purification.

**Figure 1:**
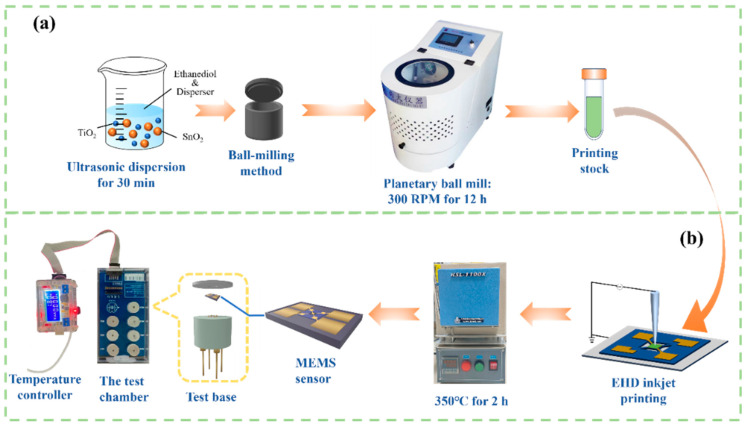
Schematic diagrams of (**a**) ball milling process; (**b**) MEMS gas sensitive chip preparation and test.

The typical process of preparation of SnO_2_-TiO_2_ slurry is shown in Figure 1a. Firstly, 0.65 g of SnO_2_ powder and 0.35 g of TiO_2_ powder are dispersed in 8.1 mL of ethylene glycol containing 0.1 g of dispersant. The obtained suspension then is ultrasonically dispersed for 30 min and transferred to a ball mill jar with a volume of 50 mL and a total of 56 grinding balls. The ball mill (QM-3SP04, Nanjing Nanda Instrument Co., Ltd., Nanjing, China) parameters are set to a frequency of 25 Hz and a milling time of 720 min. The milled slurry is then collected for later use. In this sample, the molar ratio of SnO_2_ to TiO_2_ is 1:1, and this sample is named ST1-1. Similarly, by adjusting the molar ratio of SnO_2_ to TiO_2_ to 3:1 and 1:3, the samples are denoted as ST3-1 and ST1-3, respectively. In addition, for comparison, 1 g of individual SnO_2_ and TiO_2_ were ball-milled under the same conditions, denoted as SnO_2_ and TiO_2_, respectively.

The MEMS devices were purchased from Jipai Xiangxin (Shanghai) Intelligent Technology Co., Ltd. (Shanghai, China). The structure of the MEMS micro-hotplate containing interdigital electrode, isolation, heater electrode and supporting film is shown in Figure S1. The corresponding SEM image shows that the size of the square suspended membrane (ABCD) is about 150 × 150 μm. The gas-sensitive membrane is deposited on the surface of the interdigital electrode. The preparation process of the gas-sensitive membrane is shown in Figure 1b: Firstly, the ball-milled slurry is drop-coated onto the MEMS chip functional area using EHD technology (HEDJET-H, Wuhan Huawei Technology Co., Ltd., Wuhan, China). The MEMS chip with sensing materials then is dried at room temperature and annealed at 350 °C for 2 h with the heating rate of 2 °C/min. Subsequently, the obtained MEMS sensor ages at 1.8 V for 36 h to improve its stability.

### 2.2. Characterization of Materials

The microstructure of MEMS and gas-sensitive membranes was studied using field emission scanning electron microscopy (SEM, JSM-6700F). Transmission electron microscopy and high-resolution transmission electron microscopy (TEM and HRTEM, JEM-2100 Field Emission TEM) were used to investigate the microscopic morphology and crystal structure of the composite nanomaterials. The elemental distribution of the materials was studied using the energy dispersive X-ray spectroscopy (EDS) of the JEM-F200 Field Emission Transmission Electron Microscope. X-ray diffraction (XRD, Rigaku D/MAX2200) was used to analyze the crystal structure of the product, with a scanning angle ranging from 20° to 80° at a speed of 8°/min. 

X-ray photoelectron spectroscopy (XPS, Thermofisher Nexsa) was employed to measure the chemical state of surface elements, using aluminum as the target material, operating in a high vacuum environment of 5.0 × 10^−7^ mbar, with a working voltage set at 12 kV and a filament current stabilized at 6 mA. To ensure the accuracy of the test results, the binding energy was calibrated using the C1s (284.80 eV) spectral line. The Brunauer–Emmett–Teller (BET)specific surface area of the samples was determined using an automatic specific surface area and porosity analyzer (Micromeritics ASAP 2460, USA). The pore size distribution was calculated from the adsorption branch of the nitrogen adsorption-desorption isotherm using the Barrett–Joyner–Halenda (BJH) method. Photoluminescence (PL) measurements were performed using a fluorescence spectrometer (Edinburgh FLS1000, UK) with an excitation source of a 290 nm Xe lamp. 

Oxygen temperature-programmed desorption (O_2_-TPD) tests were conducted using a temperature-programmed chemical adsorption analyzer (Hiden DECRA, UK). The test conditions were as follows: the sample was pretreated by drying at a programmed temperature ramp from room temperature to 288 °C at 10 °C/min, purged with a He gas flow (30–50 mL/min) for 1 h, cooled to 50 °C, saturated with a 10% O_2_/He mixture (30–50 mL/min) for 1 h, purged with a He gas flow (30–50 mL/min) for 1 h to remove weakly physically adsorbed O_2_ from the surface, and finally desorbed in a He atmosphere at a heating rate of 10 °C /min up to 290 °C. The desorbed gas was detected using a thermal conductivity detector (TCD).

### 2.3. Gas Sensitive Performance Test

The gas sensitivity test was performed on the electronic gas sensitivity tester smart gas sensitivity analysis system (LP-002A). One of the advantages of MEMS sensors is their low power consumption, and the relationship between power consumption and heating voltage is shown in Figure S2. For example, when the heating voltage is 2.1 V, the sensor power consumption is only 42 mW. The operating temperature of the MEMS sensor is controlled by varying the heating voltage of the heater, and the relationship between temperature and heating voltage is shown in Figure S3. During the experiment, the relative humidity of the laboratory was approximately 50%. The required amount of target gas is injected into the test chamber using a micro-injector. In the circuit for measuring gas response, the load resistance (R_L_) is connected in series with the MEMS gas sensor (Figure S4). The circuit voltage (V) is set to 5 V, and the output voltage (V_out_) is recorded 10 times per second. The relationship between MEMS sensor resistance R and V_out_ is given by Equation (1). Therefore, R can be calculated from V_out_.

R = (5 − V_out_)/V_out_ × R_L_, (1)

Sensor response is calculated using the following formula:

S = R_a_/R_g_ (2)

where R_a_ and R_g_ represent the resistance of the sensor exposed to ambient air and target gas, respectively. The response time (T_res_) and recovery time (T_rec_) refer to the time required for the response change to reach 90% of the equilibrium value after the injection and removal of the tested gas, respectively.

## 3. Results and Discussion

### 3.1. Characterization Results of Gas Sensitive Materials and Devices

**Figure 2:**
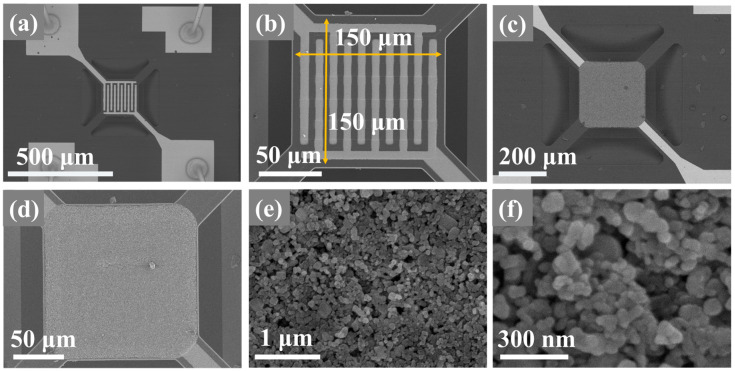
(**a**,**b**) are the SEM images of the original MEMS substrate, (**c**–**f**) are the SEM images of the gas sensitive film of ST1-1 sensor.

Figure 2a illustrates the suspended structure of the MEMS micro-hotplate, which is supported by four suspended beams in the sensing area. The area of the Pt interdigitated electrodes is 150 × 150 µm (Figure 2b). The power consumption of the MEMS sensor in working condition is approximately 42 mW, which is much lower than that of commercially available ceramic-based sensors. As observed from Figure 2c,d, the sensor prepared using EHD technology (ST1-1) forms a dense gas-sensitive film without visible cracks, completely covering the electrodes without spillage. Since gas sensitivity largely depends on the size and morphology of the sample, a uniform nanostructure can reduce measurement errors during the gas sensing process, thereby improving sensing capability and reproducibility. High-magnification SEM images of ST1-1 reveal a highly porous microstructure (Figure 2e) where nanoparticles are tightly packed together, forming a breathable microporous membrane (Figure 2f). Figure S5 shows SEM images of SnO_2_ and TiO_2_ sensors. The gas-sensitive films of both sensors also cover the functional areas of the chips well, further indicating that EHD can better control the distribution of gas-sensitive materials on the surface. However, slight cracks are visible in these two gas-sensitive films. Upon comparing them to the structure of ST1-1, it appears that the composite material aids in the formation of a uniform and dense gas-sensitive film.

**Figure 3:**
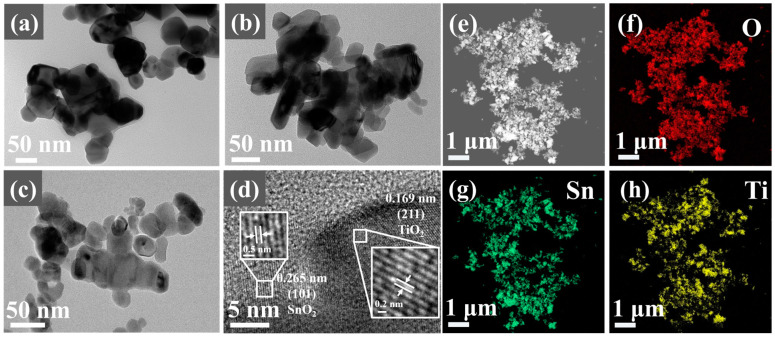
TEM images of (**a**) SnO_2_, (**b**) TiO_2_, (**c**) ST1-1; HRTEM diagram of ST1-1 (**d**); STEM image of ST1-1 (**e**); EDS mapping of ST1-1: (**f**) O, (**g**) Sn, (**h**) Ti.

Figure 3a,b present TEM images of SnO_2_ and TiO_2_, respectively. It can be observed that the particle sizes of SnO_2_ and TiO_2_ after ball milling are around tens of nanometers, but the particles are not uniform and tend to agglomerate. Figure 3c demonstrates that the particle size of the ball-milled ST1-1 composite material is mainly concentrated at about 30 nm, indicating a more uniform distribution. ST1-3 and ST3-1 also show similar results (Figure S6). Figure 3d displays an HRTEM image of the ST1-1 composite material, showing a lattice spacing of 0.265 nm for SnO_2_ (101) and 0.169 nm for TiO_2_ (211), indicating a very close spatial contact between SnO_2_ and TiO_2_. EDS results confirm that the ST1-1 composite material consists of Ti, Sn, and O, as shown in Figure 3. Figure 3a–d further demonstrate that the interface consists of SnO_2_ nanoparticles and TiO_2_ nanoparticles. The energy spectrum of the ST1-1 composite material reveals a uniform distribution of Sn and Ti throughout the entire region (Figure 3e–h and Figure S7), indicating a relatively uniform spatial distribution of SnO_2_ and TiO_2_ nanoparticles after ball milling.

**Figure 4:**
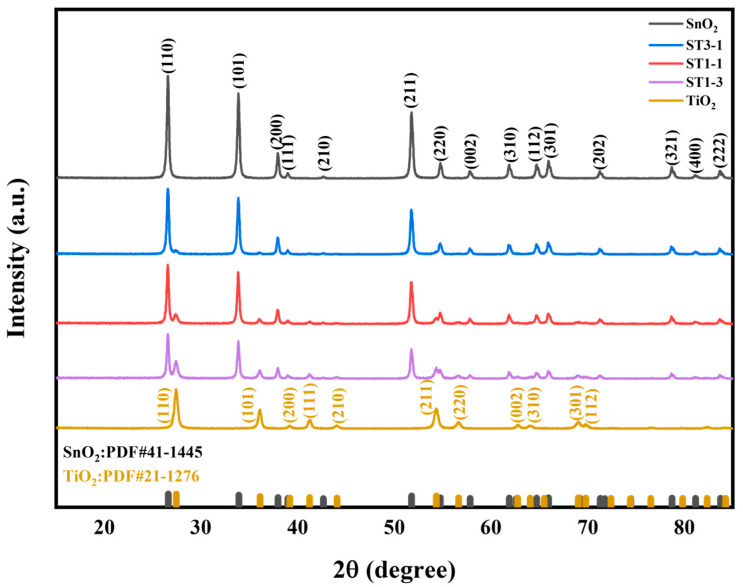
XRD patterns of SnO_2_, ST3-1, ST1-1, ST1-3, and TiO_2_.

The crystal structure and composition of the synthesized samples were analyzed by XRD, and the results are shown in Figure 4. The diffraction peaks located at 26.56°, 33.82°, 38.82°, 51.50°, and 65.42° correspond to the (100), (101), (200), (211), and (301) crystal planes of SnO_2_ (JCPDS #41-1445), respectively, indicating that the crystal form of SnO_2_ is rutile [28]. The diffraction peaks at 25.6°, 38.1°, 48.5°, 55.6°, and 62.9° correspond to the (101), (004), (200), (211), and (204) crystal planes of TiO_2_ (JCPDS #21-1276), respectively, indicating a rutile phase of TiO_2_ [30]. When SnO_2_ and TiO_2_ are mixed, the XRD spectrum mainly shows diffraction peaks of SnO_2_ and TiO_2_, and no new diffraction peaks are formed, indicating that no chemical reaction has occurred to form a new phase.

The surface properties of materials play a crucial role in determining gas sensing performance. Firstly, N_2_ adsorption–desorption isotherm tests were conducted to investigate the specific surface areas of SnO_2_, TiO_2_, and the ST1-1 composite (refer to Figure S8). As indicated in Table S1, the specific surface areas of SnO_2_, ST1-1, and TiO_2_ materials are 9.7 m^2^/g, 16.1 m^2^/g, and 25.0 m^2^/g, respectively. The specific surface area of the composite material is approximately the average of the individual oxides, suggesting that the composite material tends to neutralize the specific surface areas of the individual oxides.

X-ray photoelectron spectroscopy (XPS) was employed to investigate the surface elemental composition and valence state distribution of SnO_2_, TiO_2_, and ST1-1. In the high-resolution XPS spectrum of Sn 3d (Figure S9a), two prominent peaks can be clearly observed. Both the peaks of ST1-1 material and SnO_2_ material are centered at 486.9 eV (Sn 3d5/2) and 495.4 eV (Sn 3d3/2) without any shift, corresponding to Sn^4+^, indicating the presence of Sn in the +4 oxidation state [28,30]. In the high-resolution XPS spectrum of Ti 2p (Figure S9b), two distinct peaks are visible. The peaks of both ST1-1 and TiO_2_ materials are centered at 458.4 eV (Ti 2p3/2) and 464.4 eV (Ti 2p1/2), corresponding to Ti^4+^, demonstrating the existence of Ti in the +4 oxidation state. However, it is evident that the peaks of the ST1-1 sample are slightly shifted towards lower binding energies compared to pure TiO_2_, suggesting the formation of a portion of Ti^3+^.

**Figure 5:**
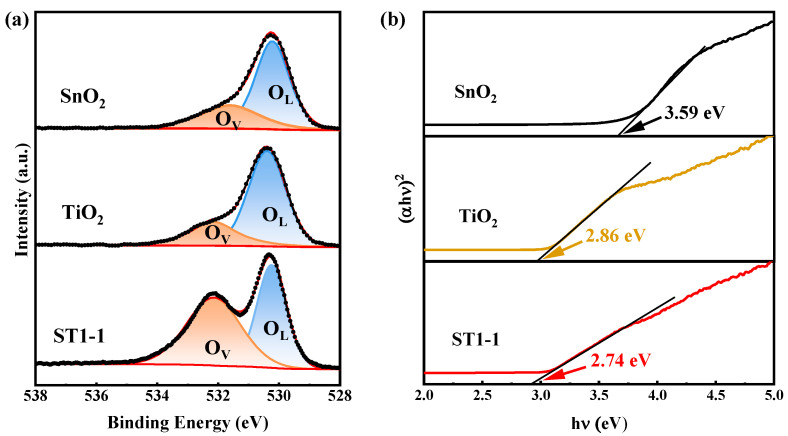
(**a**) XPS spectra of O1s; (**b**) UV-visible absorption spectra of SnO_2_, TiO_2_, and ST1-1.

The XPS spectrum of O 1s can be decomposed into two peaks, as shown in Figure 5a and Figure S10a. Generally, the peak located at 530.58 eV can be attributed to lattice oxygen (O_L_), while the peak at 532.59 eV can be attributed to oxygen defects (O_V_) on the surface. Table 1 summarizes the oxygen species of different samples. The percentage of oxygen vacancies (O_V_) in ST1-1 is 54.8%, significantly higher than 31.9% for SnO_2_, 26.6% for TiO_2_, 33.6 for ST3-1, and 30.5% for ST1-3, indicating a higher concentration of oxygen vacancies in the ST1-1 sample, which can provide more sites for oxygen molecule adsorption.

The optical absorptions of pristine SnO_2_, TiO_2_, and ST1-1 were tested using UV–Visible spectroscopy. The bandgap of a direct bandgap semiconductor can be calculated through the relationship between the Kubelka–Munk function (αhv)^2^ and photon energy (hv), as shown in Figure 5b. The direct bandgap of SnO_2_ is 3.59 eV, and the direct bandgap of TiO_2_ is 2.86 eV. The direct bandgap of the composite ST1-1 material is 2.74 eV. Obviously, compared to pristine SnO_2_ and TiO_2_, ST1-1 has a narrower bandgap, which is possibly due to the formation of defective oxygen vacancies between the valence and conduction bands. Therefore, ST1-1 contains more oxygen vacancies and exhibits a wider range of light absorption.

**Figure 6:**
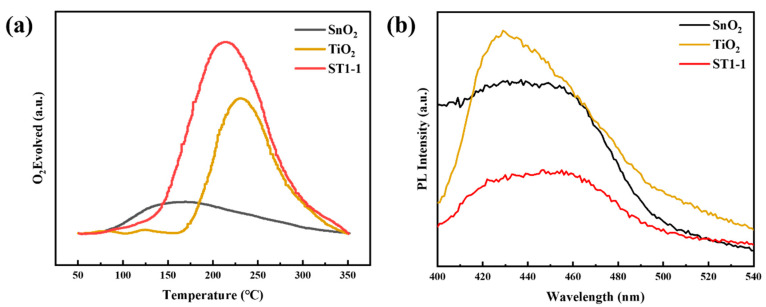
(**a**) O_2_-TPD curves of SnO_2_, ST1-1, and TiO_2_. (**b**) PL spectra of SnO_2_, ST1-1, and TiO_2_.

Further investigation into the surface active oxygen was conducted using O_2_-TPD. According to literature reports, desorption peaks below 500 °C are typically attributed to surface-adsorbed active oxygen species, including physically adsorbed oxygen (O_P_) and chemically adsorbed oxygen (O_C_), while desorption peaks above 500 °C correspond to the desorption of lattice oxygen. The results of the O_2_-TPD analysis are shown in Figure S7a. The SnO_2_ material exhibits a desorption peak at 150 °C, but the peak intensity is relatively weak. The TiO_2_ material shows a pronounced desorption peak at 230 °C, with a much stronger peak intensity compared to SnO_2_. Figure S10b indicates that ST1-3 with more TiO_2_ has stronger desorption peak compared to that of ST3-1, which is due to TiO_2_ having stronger oxygen adsorption capacity than SnO_2_, as shown in Figure 6a. The composite ST1-1 material displays a significant desorption peak at 200 °C, and its peak intensity is noticeably stronger than that of TiO_2_ and SnO_2_, consistent with the XPS results. This suggests that SnO_2_ and TiO_2_ tend to form more surface defects during the ball milling process. These findings indicate that the ST1-1 material has more adsorbed oxygen on its surface.

Photoluminescence (PL) spectroscopy was further used to analyze the charge interaction between SnO_2_ and TiO_2_. Numerous studies have shown that PL peaks originate from the recombination of excited photogenerated electrons and photogenerated holes. Therefore, a lower PL peak intensity indicates a lower recombination rate of photogenerated electrons and holes. The results are shown in Figure 6. The PL peak intensities of SnO_2_ and TiO_2_ alone are relatively strong, but the PL peak intensity significantly decreases when they are combined, indicating the lowest peak intensity for the composite ST1-1. As for the SnO_2_-TiO_2_ sample (Figure S10c), the sample of ST1-3 with higher amount of TiO_2_ own stronger PL peak intensity, which is possibly due to TiO_2_ having a stronger PL peak intensity, as shown in Figure 6b. Above results suggest that electron transfer may occur after the combination of SnO_2_ and TiO_2_, thereby reducing the recombination rate of photogenerated electron–hole pairs.

### 3.2. Gas Sensitive Property

**Figure 7:**
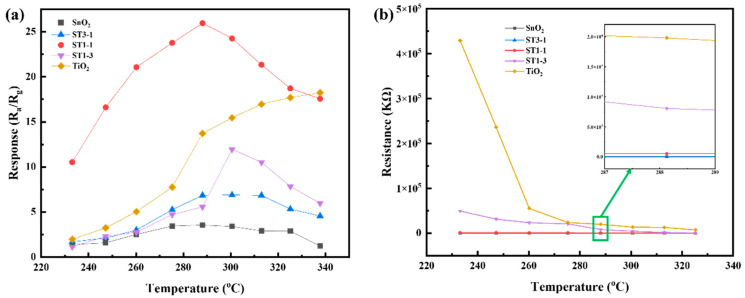
(**a**) Temperature–response curves; (**b**) temperature–initial resistance curves for different samples.

In the process of gas sensitivity performance testing, operating temperature has a significant impact on the reactions occurring on the surface and is an important parameter for gas-sensitive materials. Therefore, the effect of operating temperature on gas sensitivity performance was first studied. The response values of different sensors to 50 ppm C_2_H_5_OH were tested within a temperature range of 230 °C to 330 °C. The test environment was at room temperature with 50% relative humidity. The test results are shown in Figure 7a. As the operating temperature increases, except for the TiO_2_ sensor, the response of other sensors shows a volcanic trend that first increases and then decreases. For SnO_2_, its response values are relatively low throughout the entire tested temperature range. At the optimal operating temperature of 288 °C, the response value is 3.6. In contrast, the TiO_2_ sensor has a response value of 13.8 at the same temperature, indicating a higher response to ethanol. However, after combining the two materials, the response values have significantly improved compared to SnO_2_. Specifically, the response values of the ST3-1 and ST1-3 sensors at their optimal operating temperatures are 6.9 and 5.6, respectively, which are higher than that of SnO_2_ but smaller than that of the TiO_2_ sensor at the corresponding temperature. Nonetheless, when the molar ratio of SnO_2_ to TiO_2_ is 1:1, the ST1-1 sensor achieves a remarkable response value of 25.6 at the optimal operating temperature of 288 °C. This represents 7.2-fold and 1.8-fold increases compared to the response values of the individual SnO_2_ and TiO_2_ sensors, respectively. This significant enhancement indicates that combining SnO_2_ and TiO_2_ in an appropriate ratio can greatly improve the sensor’s response performance.

Changes in gas-sensitive resistance were also observed when varying the operating temperature, and the results are shown in Figure 7b. At 233 °C, the TiO_2_ sensor exhibits the highest resistance, approximately 429 MΩ, while the SnO_2_ sensor has the lowest resistance of approximately 63 KΩ. When the two materials are combined, the resistance decreases as the amount of SnO_2_ added increases. As the temperature rises, the R_a_ of all samples decreases. Among them, the TiO_2_ sensor shows a significant decrease in resistance with increasing temperature, while the resistance of SnO_2_ remains notably lower throughout the entire tested temperature range. At the optimal operating temperature of 288 °C, the resistances of the R_a_(SnO_2_), R_a_(ST3-1), R_a_(ST1-1), R_a_(ST1-3), and R_a_(TiO_2_) sensors are 25 KΩ, 33 KΩ, 516 KΩ, 8060 KΩ, and 19,800 KΩ, respectively. As the TiO_2_ content increases, the resistance gradually increases. Additionally, it’s worth noting that high resistance can pose challenges for device circuit design. Therefore, combining SnO_2_ and TiO_2_ is beneficial for obtaining more practical resistance values.

**Figure 8:**
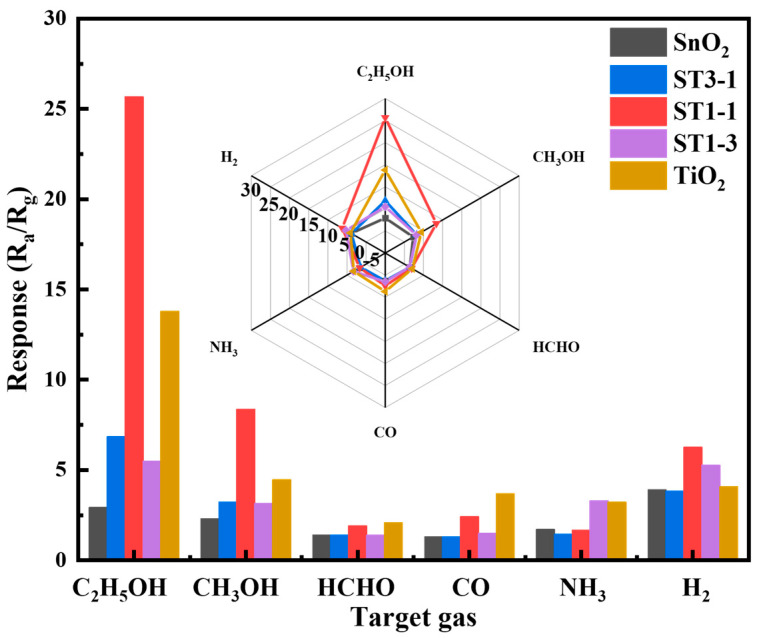
Selective testing of five gas sensors.

Selectivity is a crucial performance indicator for gas sensors in practical applications, as it determines the sensor’s ability to recognize target gases and resist interference. Figure 8 shows the response values of different sensors under the influence of 50 ppm ethanol and common reducing interference gases such as CH_3_OH, HCHO, CO, NH_3_, and H_2_ at a working temperature of 288 °C. As can be seen from the figure, under the same conditions, the responses of SnO_2_ sensor to CH_3_OH, HCHO, CO, NH_3_, and H_2_ are 2.3, 1.4, 1.3, 1.7, and 3.9, respectively, while the response to C_2_H_5_OH is 2.9. This indicates that the SnO_2_ sensor does not have good selectivity for ethanol. The responses of TiO_2_ sensor to CH_3_OH, HCHO, CO, NH_3_, and H_2_ are 4.5, 2.1, 3.7, 3.2, and 4.1, respectively, while the response to C_2_H_5_OH is 13.8. This suggests that the TiO_2_ sensor has better selectivity for ethanol. When the two materials are combined, the selectivity of ST1-3 and ST3-1 does not significantly improve. The responses to different gases are low. However, the ST1-1 sensor shows a significant enhancement in responsiveness to ethanol. Its responses to CH_3_OH, HCHO, CO, NH_3_, and H_2_ are 8.3, 1.9, 2.4, 1.6, and 6.2, respectively, while the response to C_2_H_5_OH is 25.6, which is significantly higher than that for other gases. These results indicate that the ST1-1 sensor combines the advantages of both metal oxides, improving selectivity for ethanol vapor while lowering the optimal operating temperature. 

**Figure 9:**
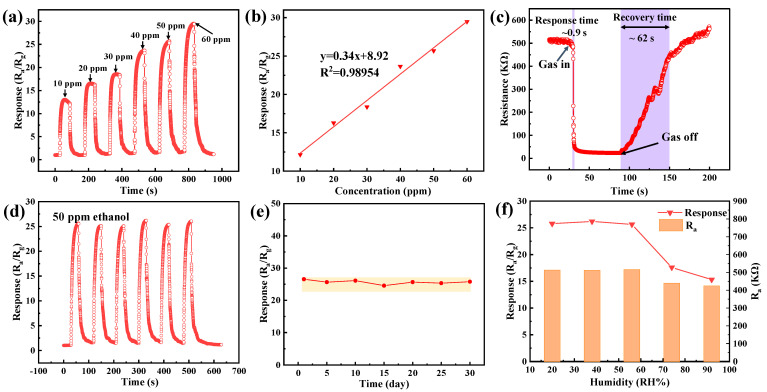
Gas sensing performance of the ST1-1 sensor. (**a**) Response to 10 –60 ppm of ethanol and corresponding (**b**) linear fitting curve; (**c**) making their selectivity towards 50 ppm ethanol; (**d**) repeatability testing in 50 ppm of ethanol; (**e**) long-term stability in 30 days; and the (**f**) The effect of humidity on response and initial resistance in 50 ppm of ethanol.

The sensing performance of the ST1-1 sensor to ethanol at a working temperature of 288 °C was further investigated. Firstly, the response and recovery of the ST1-1 sensor to different concentrations (10–60 ppm) of ethanol were studied. The results, shown in Figure 9a, indicate that the response value increases linearly with increasing gas concentration from 10 ppm to 60 ppm, with a fitted line slope of 0.34 (Figure 9b). The response speed and recovery speed of the MEMS sensor can be evaluated by response time and recovery time. As shown in Figure 9c, the response time of the ST1-1 sensor at 288 °C is approximately 11.8 s, and the recovery time is approximately 60.1 s, indicating relatively fast response and recovery speeds of the ST1-1. The stability of the ST1-1 sensor is demonstrated in Figure 9d. There is no significant difference in the response values during six cycles of testing under 50 ppm ethanol conditions, indicating excellent signal reproducibility and short-term repeatability of the ST1-1 sensor. Figure 9e shows the long-term stability of the ST1-1 sensor tested over 30 days under the same environmental conditions with 50 ppm ethanol. No significant drift or downward trend was observed within 30 days, indicating good long-term stability. Additionally, the effects of humidity on sensing performance and initial resistance were studied. The changes in the response and initial resistance of the ST1-1 sensor are shown in Figure 9f, with an ambient temperature of 25 °C and a relative humidity (RH) range of 20–90%. Both parameters show small fluctuations in the low RH range of 25–55%, a significant decrease in the 55–75% range, and a slow decrease in the 75–90% range, requiring temperature drift correction. Consistency is also one of the important indicators of sensor performance. Figure S11 shows that three MEMS gas sensors prepared under the same conditions respond almost identically to 50 ppm C_2_H_5_OH, illustrating that EHD technology provides a reliable and effective method for preparing gas sensors with excellent consistency.

We also compared the comprehensive sensing performance of our ST1-1 sensor with MEMS devices reported in the literature. As shown in Table 2, the ST1-1 sensor exhibits a higher response and lower power consumption compared to most reported materials.

### 3.3. Discussion of Gas-Sensitive Mechanism

The sensing mechanism of MOS gas sensors is related to the resistance changes caused by gas adsorption and desorption. The gas-sensing performance is closely associated with surface adsorbed oxygen, and variations in the amount of adsorbed oxygen can lead to changes the resistance of materials, enabling gas detection [39,40].

In this study, SnO_2_ and TiO_2_ were combined using a ball milling process. During ball milling, differences in the material properties of SnO_2_ and TiO_2_ can lead to the creation of surface defects. XPS results indicate that ST1-1 has more oxygen defects than SnO_2_ and TiO_2_ (the oxygen vacancy contents of SnO_2_, TiO_2_, and ST1-1 are 31.9%, 26.6%, and 54.8%, respectively), which is also supported by UV–Visible absorption spectroscopy. Many studies have shown that surface oxygen defects can significantly promote the formation of surface-active oxygen. The O_2_-TPD test results further demonstrate that the amount of adsorbed oxygen on the composite gas-sensing material increases significantly, playing a crucial role in enhancing the gas-sensing performance of ST1-1 [41].

It is well known that the work functions of TiO_2_ and SnO_2_ are 5.58 eV and 4.9 eV, respectively. When TiO_2_ comes into contact with SnO_2_, significant electron transfer occurs at their interface, with electrons moving from SnO_2_ to TiO_2_. This charge transfer alters the electronic distribution at the interface, optimizing the surface electronic structure of the composite material. PL spectroscopy (Figure S6b) provides evidence of this electron transfer process. The interfacial charge transfer creates an electron depletion layer on the SnO_2_ side and further bends the energy bands, resulting in a higher resistance for the sensing material compared to a pure SnO_2_ sensor, consistent with our observations (Figure 6b). Due to the different electronic affinities and chemical properties of SnO_2_ and TiO_2_, interfacial charge transfer can modulate the surface chemical state of the composite material. This enhances the selectivity of the composite material towards specific gas molecules, enabling accurate identification and detection of target gases in complex gaseous environments.

According to the literature, the adsorption of ethanol on the SnO_2_-TiO_2_ surface actually involves both physical and chemical adsorption processes. Firstly, at temperatures below 100 °C, oxygen in the air is physically adsorbed onto the SnO_2_-TiO_2_ surface, where it captures electrons to form chemically adsorbed O_2_^−^ (ads), as described by Formula (3):

O_2 (gas)_ + e^−^ → O_2_^−^ _(ads)_, (T < 100 °C) (3)

At room temperature, the reaction rate of this process is very slow. However, as the temperature rises, the rate of chemical adsorption of oxygen increases, and O_2_^−^ (ads) can be transformed into more stable O^−^ (ads) and O^2−^ (ads) through Formulas (4) and (5) [42,43]:

O_2_^−^ _(ads)_ + e^−^ → 2O^−^ _(ads)_, (100 °C < T < 300 °C) (4)

O^−^ _(ads)_ + e^−^ → O^2−^ _(ads)_, (T > 300 °C) (5)

**Figure 10:**
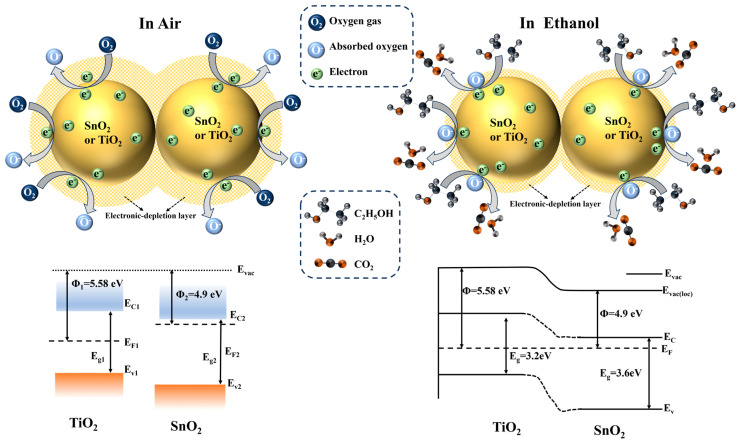
Gas sensing mechanism diagram of ST1−1 sensor.

These chemically adsorbed oxygen ions create an electron depletion layer on the SnO_2_-TiO_2_ surface, resulting in an increase in the resistance of SnO_2_-TiO_2_. When exposed to an ethanol atmosphere, ethanol molecules adsorb onto the SnO_2_-TiO_2_ surface and undergo a redox reaction with the chemically adsorbed O^−^ (ads), as described by Formula (6), Here, ethanol was oxidized to form CO_2_ and H_2_O [29,44]. This reaction releases electrons back into the conduction band of SnO_2_-TiO_2_, causing the potential barrier to decrease, the depletion layer to narrow, and the resistance to decrease. The chemical adsorption process of ethanol molecules on the SnO_2_-TiO_2_ surface is illustrated in Figure 10.

C_2_H_5_OH _(gas)_ + 6O^−^ _(ads)_ → 2CO_2_ + 3H_2_O + 6e^−^ (6)

Therefore, compared to single SnO_2_, TiO_2_, ST3-1, and ST1-3, ST1-1 exhibit stronger adsorption for oxygen and ethanol. In particular, the reactive oxygen on the surface facilitates the reaction with ethanol, resulting in changes in resistance and achieving efficient ethanol response. Additionally, the selective adsorption and oxidation of ethanol over SnO_2_ and TiO_2_ facilitate the selective detection of ethanol among different gases [7,8,24].

## 4. Conclusions

In summary, we report a SnO_2_-TiO_2_-nanocomposite-based MEMS sensor for ethanol detection. Gas-sensitive materials with different SnO_2_-TiO_2_ composite ratios were prepared using a mechanical ball milling method, resulting in three nanocomposite films with varying molar ratios of SnO_2_-TiO_2_. SEM images of the films revealed that combining the two materials contributes to a more uniform and stable gas-sensitive film. Studies on the ethanol gas sensitivity of sensors with different composite ratios showed that when the molar ratio of SnO_2_ to TiO_2_ is 1:1, the resulting composite slurry exhibits the best response to ethanol. At a working temperature of 288 °C, the response to 50 ppm ethanol is 25.6, which is 7.2 times that of pure SnO_2_ and 1.8 times that of pure TiO_2_. Comprehensive testing of these gas sensors revealed that the ST1-1 gas sensor exhibits high selectivity and good gas response characteristics in a mixed gas environment (C_2_H_5_OH, CH_3_OH, HCHO, CO, NH_3_, H_2_). This is mainly attributed to the chemical and electronic effects of the composite material, which bring about changes in surface-active oxygen and material resistance. By adjusting the composition, structure, and interface properties of the composite material, the number and activity of surface-active oxygen, as well as the material’s resistance, can be optimized to meet the needs of specific applications.

## Figures and Tables

**Table 1:** Central positions and relative percentages of O_L_ and O_V_.

Sample	Oxygen Species	Binding Energy (eV)	Relative Percentage (%)
SnO_2_	O_L_	530.2	68.1
	O_V_	531.6	31.9
TiO_2_	O_L_	530.4	73.4
	O_V_	532.2	26.6
ST1-1	O_L_	530.3	45.2
	O_V_	532.2	54.8
ST3-1	O_L_	530.2	66.4
	O_V_	531.4	33.6
ST1-3	O_L_	530.5	69.5
	O_V_	532.2	30.5

**Table 2:** A comparison of sensing performance between current work and previously reported results over MOS-based MEMS sensor.

Materials	Op. Tem. (°C)	Response	T_res_/T_rec_ (s)	Work Power (mW)	Ref.
SnO_2_-TiO_2_	288	25.6 (50 ppm)	11.8/20.6 (50 ppm)	42	This work
Au–SnO_2_	400	10 (100 ppm)	248/538 (100 ppm)	55	[9]
Pd/SnO_2_:NiO	350	13.3 (50 ppm)	-/-	-	[31]
SnO_2_/MXene	230	9.2 (50 ppm)	14/26 (10 ppm)	-	[32]
SnO_2_ Nanosheets	300	4.1 (500 ppm)	-/-	-	[33]
TiO_2_/SnO_2_	260	7.54 (50 ppm)	33/298 (50 ppm)	-	[34]
SnO_2_:NiO network	300	9 (50 ppm)	-/-	-	[35]
Pt/TiO_2_/SnO_2_	130	14.58 (25 ppm)	20/973 (25 ppm)	-	[36]
Pd/SnO_2_	300	-	1.5/18 (50 ppm)	45	[37]
Ag@SnO_2_	RT	2.24 (200 ppm)	34/68 (200 ppm)	-	[38]

## Data Availability

The data used in this study are available from the corresponding authors upon reasonable request.

## Supplementary Materials

The following supporting information can be downloaded at: https://www.mdpi.com/article/10.3390/s24154866/s1, Figure S1: The spatial structure of MEMS micro-hotplate. Figure S2: The relationship between power consumption and heating voltage; Figure S3: The relationship between temperature and heating voltage; Figure S4: Circuit diagram of MEMS sensor; Figure S5: The SEM images of the prepared (a–d) SnO_2_ gas sensor gas sensitive film and (e–h) TiO_2_ gas sensor film; Figure S6: TEM images of (a) ST3-1 and (b) ST1-3; Figure S7: STEM image and EDS mapping images of ST1-1 composite: (a) STEM image, (b) O, (c) Sn, (d) Ti; Figure S8: (a) N_2_ adsorption-desorption isotherm tests and (b) average pore sizes of different samples; Figure S9: XPS spectra: (a) Sn 3d for ST1-1 and SnO_2_, (b) Ti 2p for ST1-1 and TiO_2_; Figure S10: (a) O1s-XPS spectra, (b) O2-TPD curves, and (c) PL spectra of ST3-1 and ST1-3; Figure S11: Consistency of ST1-1 gas sensors; Table S1: Specific surface area and average pore size of SnO_2_, ST1-1, and TiO_2_.
