# Supplementary material for: Electrohydrodynamic-Jet-Printed SnO2-TiO2-Composite-Based Microelectromechanical Systems Sensor with Enhanced Ethanol Detection
Source: Sensors (Basel). 2024 Jul 26;24(15):4866. doi: 10.3390/s24154866 (PMC11314699; doi:10.3390/s24154866)
Supplement: Supplementary file 1 [file sensors-24-04866-s001.zip › sensors-3049839-supplementary.pdf]

Supplementary Materials

Electrohydrodynamic jet printed SnO_2-TiO_2 composite based microelectromechanical systems sensor with enhanced ethanol detection

Danyang Wang [1], Dongqi Yu [1], Menghan Xu[1,2], Xue Chen [1], Jilin Gu [1,*] and Lei Huang [2,*]

[1] School of Physics and Electronic Technology, Liaoning Normal University, Dalian 116029, P. R. China; W_danyang@126.com(D.W); 1430155786@qq.com(X.C)

[2] Research Center of Nano Science and Technology, College of Sciences, Shanghai University, Shanghai 200444, P. R. China;

* Corresponding authors' email: Jilin Gu, gujilin@lnnu.edu.cn; Lei Huang, leihuang@shu.edu.cn.

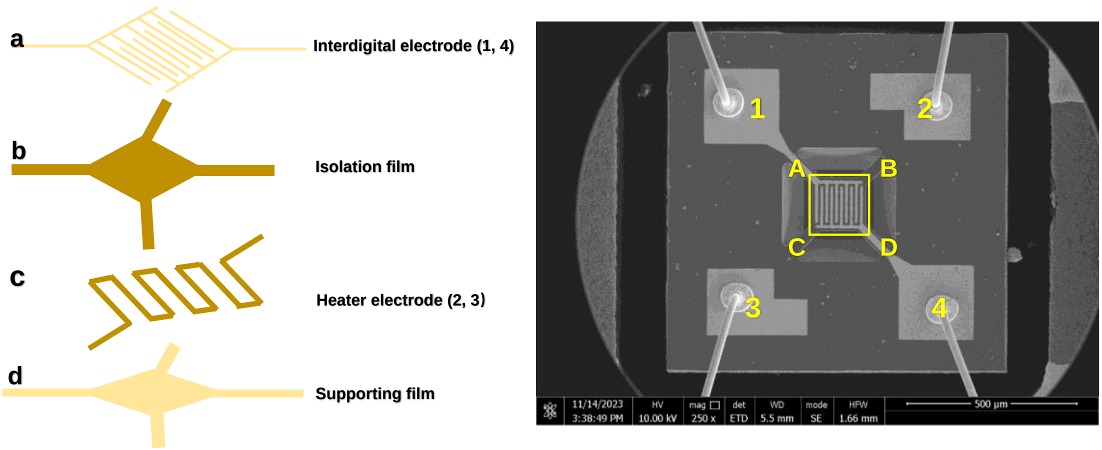

Figure S1 The spatial structure of MEMS micro-hotplate containing (a) interdigital electrode, (b) isolation, (c) heater electrode and (d) supporting film. The SEM image of MEMS micro-hotplates.

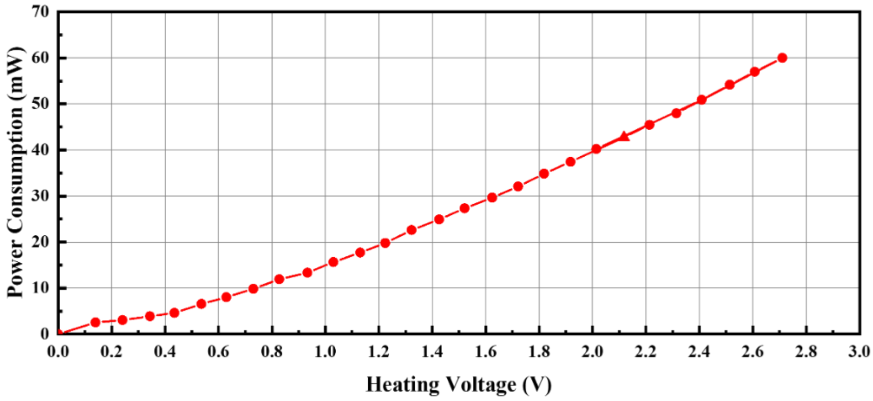

Figure S2 The relationship between power consumption and heating voltage

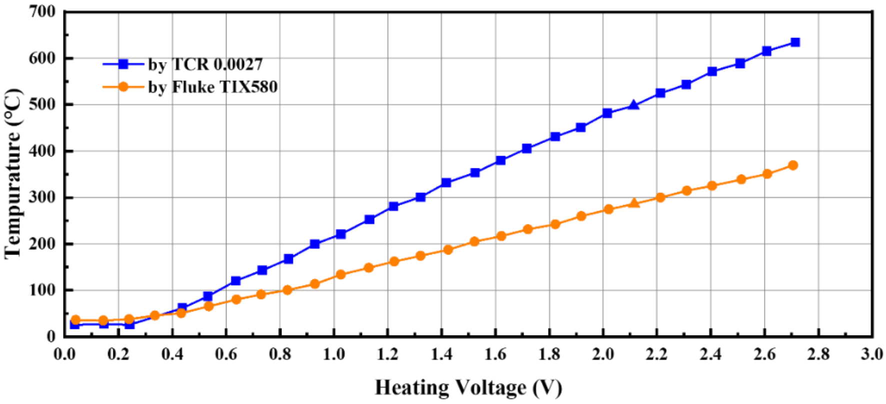

Figure S3 The relationship between temperature and heating voltage

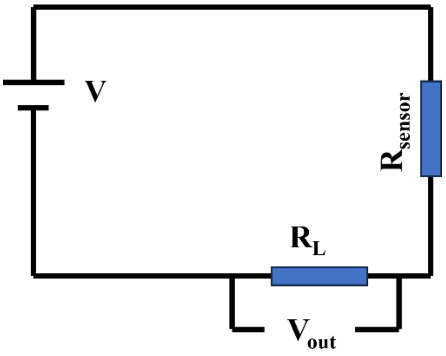

V: Circuit voltage (5 V)

V_{out}: Output voltage

R_L: Load resistance

R_{sensor}: Sample resistance

Figure S4 Circuit diagram of MEMS sensor

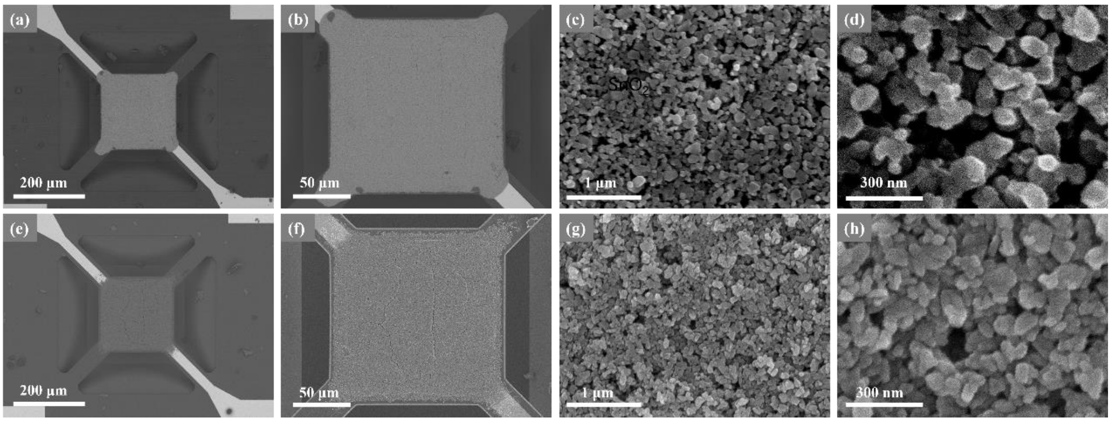

Figure S5 The SEM images of the prepared (a-d) SnO_2 gas sensor gas sensitive film and (e-h) TiO_2 gas sensor film.

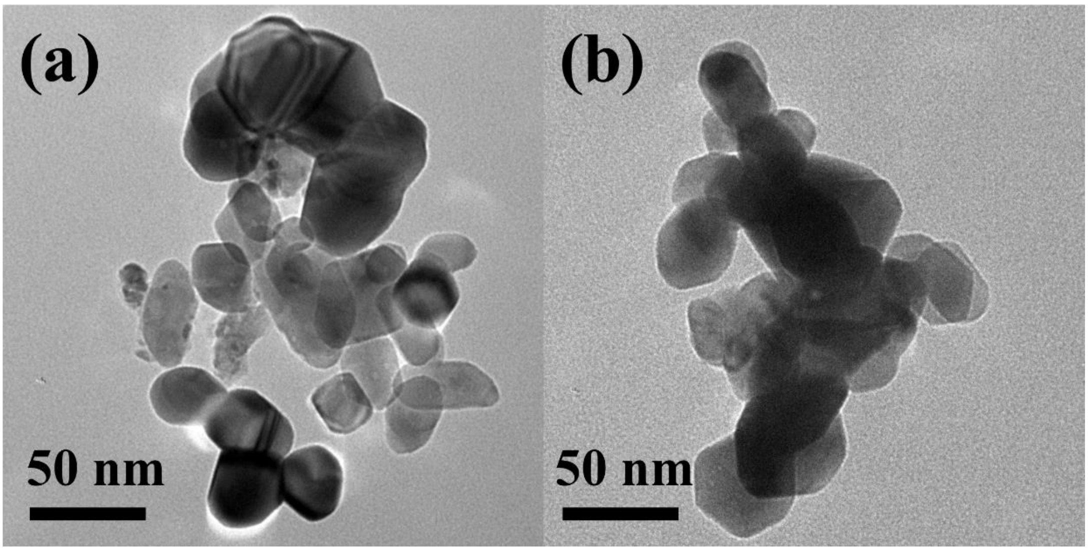

Figure S6 TEM images of (a)ST3-1 and (b)ST1-3

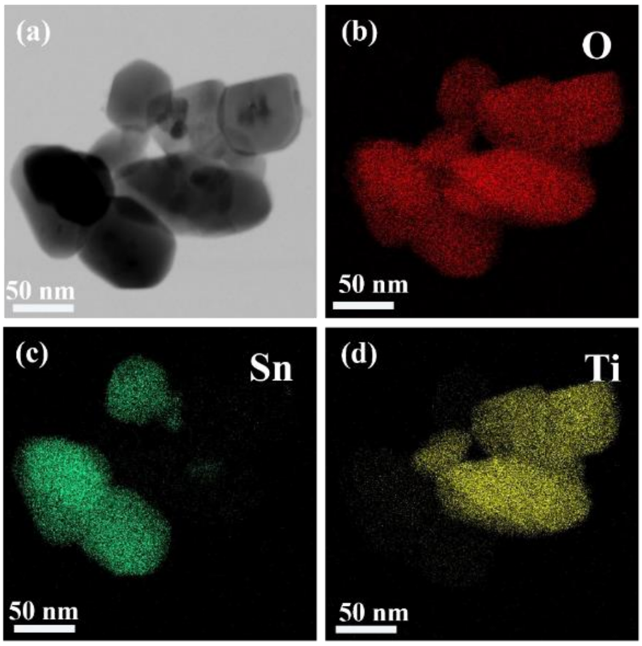

Figure S7 STEM image and EDS mapping images of ST1-1 composite: (a) STEM image, (b)O, (c)Sn, (d)Ti

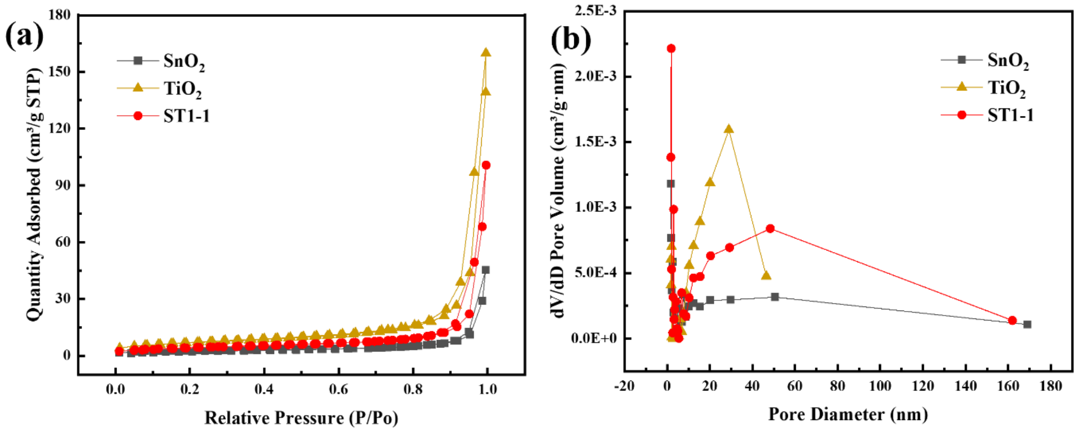

Figure S8 (a) N_2 adsorption-desorption isotherm tests and (b) pore information of different samples

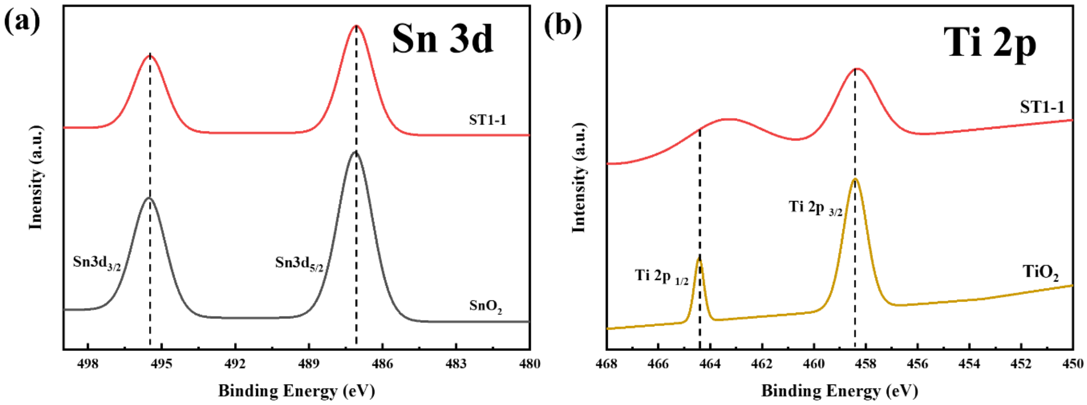

Figure S9 XPS spectra: (a) Sn 3d for ST1-1 and SnO_2, (b)Ti 2p for ST1-1 and TiO_2

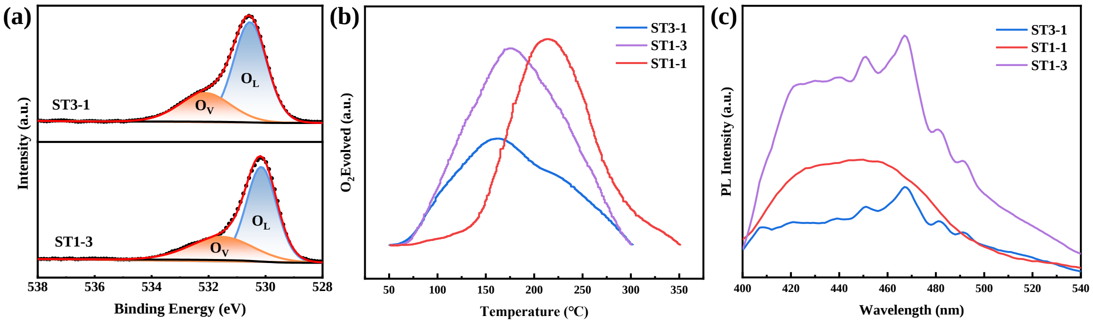

Figure S10 (a) O1s-XPS spectra, (b) O_2-TPD curves and (c) PL spectra of ST3-1 and ST1-3

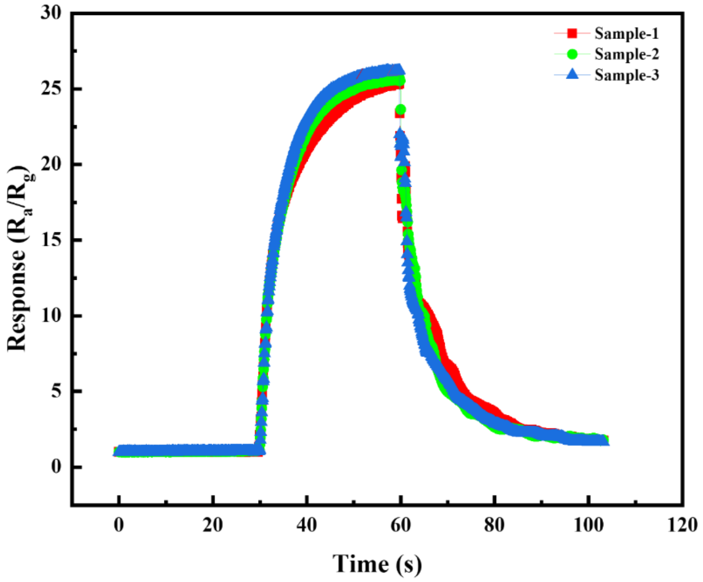

Figure S11 Consistency of ST1-1 gas sensors

Table S1: Specific surface area and average pore size of SnO_2, ST1-1 and TiO_2

Sample	BET (m^2/g)	Pore Diameter (nm)
SnO_2	9.7	30.1
ST1-1	16.1	38.7
TiO_2	25.0	39.6
